# Sound Source Distance Estimation Using Deep Learning: An Image Classification Approach

**DOI:** 10.3390/s20010172

**Published:** 2019-12-27

**Authors:** Mariam Yiwere, Eun Joo Rhee

**Affiliations:** Department of Computer Engineering, Hanbat National University, Daejeon 34158, Korea

**Keywords:** sound source distance estimation, log-scaled mel spectrogram, deep learning, convolutional recurrent neural network

## Abstract

This paper presents a sound source distance estimation (SSDE) method using a convolutional recurrent neural network (CRNN). We approach the sound source distance estimation task as an image classification problem, and we aim to classify a given audio signal into one of three predefined distance classes—one meter, two meters, and three meters—irrespective of its orientation angle. For the purpose of training, we create a dataset by recording audio signals at the three different distances and three angles in different rooms. The CRNN is trained using time-frequency representations of the audio signals. Specifically, we transform the audio signals into log-scaled mel spectrograms, allowing the convolutional layers to extract the appropriate features required for the classification. When trained and tested with combined datasets from all rooms, the proposed model exhibits high classification accuracies; however, training and testing the model in separate rooms results in lower accuracies, indicating that further study is required to improve the method’s generalization ability. Our experimental results demonstrate that it is possible to estimate sound source distances in known environments by classification using the log-scaled mel spectrogram.

## 1. Introduction

Sound source distance estimation is a process that determines the spatial length between a sound source and a receiver in a given area. It determines the location of a sound source in terms of distance. It can be used to complement the well-known sound source localization (direction and elevation only) methods [1,2,3,4,5,6,7,8,9] to enhance their localization accuracy and improve their effectiveness. For example, in a security breach situation where there is a gunshot, an intelligent surveillance system [10] can detect the sound of the gunshot, localize its azimuth, and estimate its distance in addition. This could provide information to assist the police and other emergency personnel by reducing their emergency response time [11]. Such a method could also be applicable in human–robot interaction systems [12], service robots [13], intelligent hearing aids [14], smart homes [15], etc. 

Although the location of a sound source entails the azimuth (horizontal angle), elevation, and distance [16], previously proposed sound source localization methods mostly focus on either the azimuth aspect only [1,2,3,4,5] or the azimuth and elevation aspects only [7,8,9], without distance. The distance estimation aspect of sound source localization has received relatively less attention in the scientific research community compared to the azimuth and elevation [16]. However, considering its potential usefulness in the face of rapidly advancing technology and artificial intelligence (AI), it is imperative for this topic to be studied in order to develop useful solutions.

In the past few years, researchers have depended upon hand-crafted features to estimate sound source distances. Among the proposed features that have been used to tackle the sound source distance problem is the room impulse response (RIR) [17]. For example, Bronkhorst [18] proposed a method for distance perception in rooms by applying information about the RIR. Another popular feature was the ratio of the energy of the signal received directly from the source to that of the signal that reflects from surfaces, which is a feature known as the direct-to-reverberant-ratio (DRR) [19]. Lu et al. [20] suggested a binaural distance estimation method using DRR, which they computed by first estimating the sound source’s direction and then removing the energy of the sound in that region in order to identify the reverberant signal. Rodemann [21] investigated several audio cues, including the interaural intensity difference (IID), interaural temporal difference (ITD), sound amplitude, and spectral characteristics, discovering that in certain circumstances, mean signal amplitude and binaural cues work well in estimating sound source distances. Another method for estimating the distance of single-channel audio signals was proposed by Honda et al. [22], who used the phase interference between observed and pseudo-observed signal waves to estimate the signal’s distance.

Other researchers used traditional machine learning approaches, which also required hand-crafted features. For example, Vesa [23] proposed a distance-dependent feature in the form of magnitude squared coherence (MSC) for binaural sound source distance learning using Gaussian mixture models (GMMs). This feature was motivated by the fact that an increase in the source to receiver distance leads to a decrease in the correlation between signal channels. Another method proposed by Georganti et al. [24] uses a novel feature known as binaural signal magnitude difference standard deviation (BSMD-STD) in addition to statistical properties of binaural sounds to estimate the source distance. They also trained support vector machines (SVMs) and GMMs for the estimation. While their proposed BSMD-STD feature proved to be effective in detecting the distance of sound source at a 0° orientation, its performance was significantly reduced for sound sources in other orientations, and it requires many other statistical values in other to function accurately. Georganti et al. [25] also suggested the use of statistical parameters of speech source excitation signals for the distance estimation of single-channel audio signals. Niu et al. [26] used supervised machine learning methods including a feed-forward neural network (FNN) and an SVM to perform source localization in an ocean waveguide. They used normalized sample covariance matrices, which included both amplitude and phase information as their input features, and they showed that the classification approach to source localization performed better than multi-field processing (MFP) and regression approaches. In addition, Brendel and Kellermann [27] proposed a learning-based approach that trains a GMM to estimate source–microphone distances in a known environment. Their method relies on the estimation of the coherent-to-diffuse power ratio, which indicates the amount of reverberation present in each frequency bin of the signal.

In recent years, many deep learning-based sound source localization methods have been proposed due to the popularity and remarkable performance of deep neural networks [3,4,5,6,9]; however, deep learning-based sound source distance estimation methods are comparatively scarce. In the area of source localization in ocean or water waveguides, researchers [28,29] have applied deep learning methods to successfully determine the range of sound pressure. Yiwere and Rhee [30] proposed a deep learning-based method for the joint estimation of sound source azimuths and distances using cross-correlation coefficients as input features; this study seeks to focus on the distance estimation aspect and improve the accuracy by exploring a different type of input feature.

Through a survey of the existing literature on the topic of sound source distance estimation, we found that a main challenge in sound source distance estimation is the identification of suitable distance-dependent features. While some researchers [22,23,24,25,26,27] have come up with hand-crafted features that are capable of estimating distance, their extraction processes are either complex or tedious. In addition, for a distance estimation or source localization algorithm, accuracy is a paramount factor; however, the accuracies of existing distance estimation methods are still far from perfect and must be improved. To solve the above-mentioned problems, this study proposes a deep learning-based sound source distance estimation method. The method trains a convolutional recurrent neural network (CRNN) [31,32] using log-scaled mel spectrograms [33] extracted from single-channel audio signals as input features. The transformation of the audio signals into images allows the convolution layers of the network to automatically extract distance-dependent features from the audio signals; therefore, this approach comes with the advantage of eliminating the need for excessive hand-crafted feature engineering, in addition to achieving high classification accuracy. We create an audio dataset for the purpose of sound source distance estimation, using a two-microphone array. However, in this method, we use only one channel signal. In addition, we use a publicly available room impulse response dataset convolved with speech data to evaluate the generalization ability of the proposed method. 

As mentioned above, the proposed method could be incorporated into any application that could benefit from the knowledge of a sound source’s position in the environment. The most obvious example being sound source localization applications, which could leverage distance estimation to make a more accurate and complete estimation of a sound source’s position. Similar to the work of Georganti et al. [25], this method is typically applicable in applications for distributed ambient telephone systems [34]. In such a system, the ability to select the microphone closest to the human speaker could help enhance the signal-to-noise ratio of the received speech signal prior to transmission. 

The organization of this paper is as follows. Section 2 presents the materials and methods, which includes the data collection and transformation processes, as well as a description of the deep learning model architecture. Section 3 presents the experiments and results, and the results are discussed in Section 4. Finally, Section 5 summarizes the study in the conclusion. 

## 2. Materials and Methods

In this section, a description of the first part of the study is provided. This includes data collection and transformation of the audio signals into spectrograms. Unlike other audio processing tasks such as sound event classification [35,36] and automatic speech recognition [37], this study requires the collection and labeling of audio data as described below. We recorded audio data by playing speech signals from a loudspeaker [38]. The speech signals were taken from the Edinburgh University Speech Timing Archive and Corpus of English (EUSTACE) downloadable corpus [39], which is a free database provided by the Center for Speech Technology Research at the University of Edinburgh.

### 2.1. Data Collection

**Figure 1:**
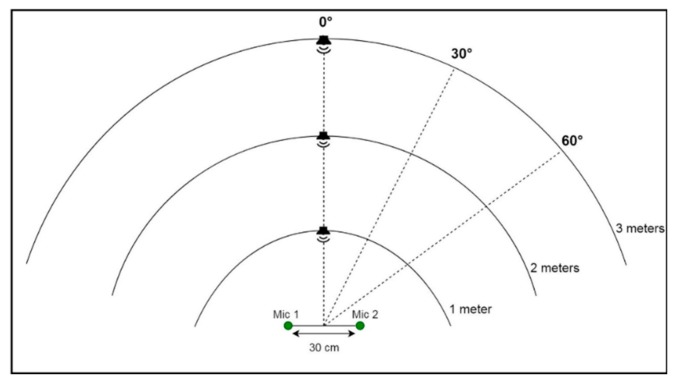
Training data recording positions.

To set up the system for recording, we used two microphones positioned at a distance of 30 cm apart. The speech signals were played from a loudspeaker, which was positioned at three different distances in front of and facing the microphone setup (i.e., at the 0° orientation); specifically at one-meter, two-meter, and three-meter positions. The volume of the speaker was varied during the recording to avoid a clear distinction between the three distance classes based on the volume. The microphones were connected to a TASCAM US 4 × 4 audio interface for analogue-to-digital conversion of the signals, and we used the PortAudio library [40] to capture the sound at a sampling rate of 44.1 kHz. When recording began, the algorithm saved one-second-long clips of the sound into the computer’s drive. The process was repeated at two other orientation angles, i.e., 30°, and 60°, as shown in Figure 1. The recording was done in three different rooms.

**Figure 2:**
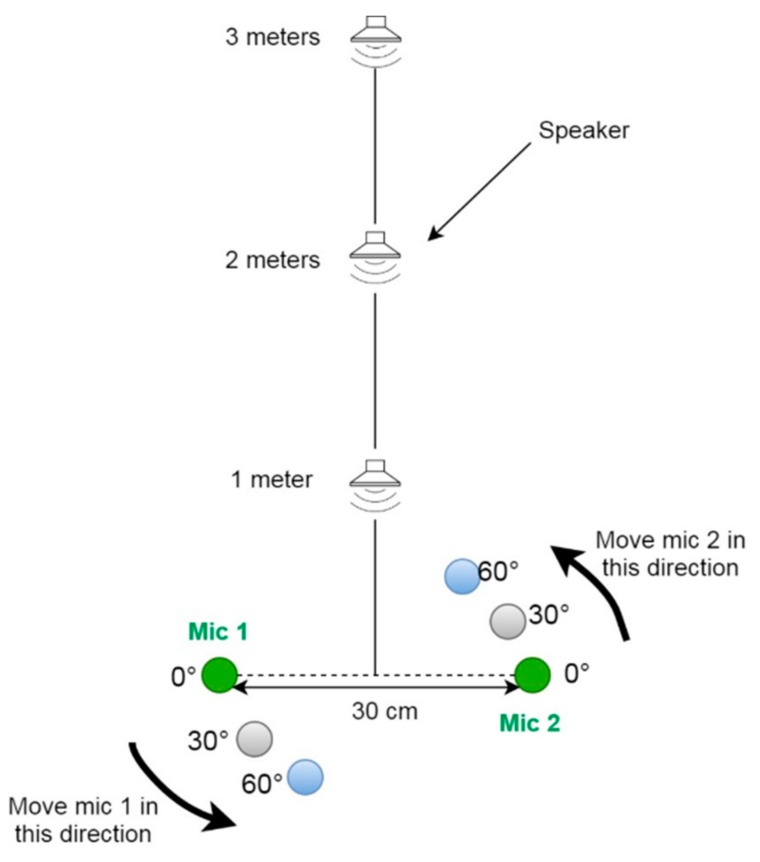
Illustration of the rotated microphone approach.

Figure 1 shows all the nine recording positions at which the sound source should be placed. This implies that after all the recordings for a particular orientation are taken, the sound source should be shifted to a different orientation. However, in certain rooms, this might be impossible if there is not enough space, in which case an alternative approach is taken, by keeping the sound source at the 0° orientation and rotating the microphone array for the other two orientations. Figure 2 illustrates the alternative approach for recording, where the microphones are rotated. 

**Figure 3:**
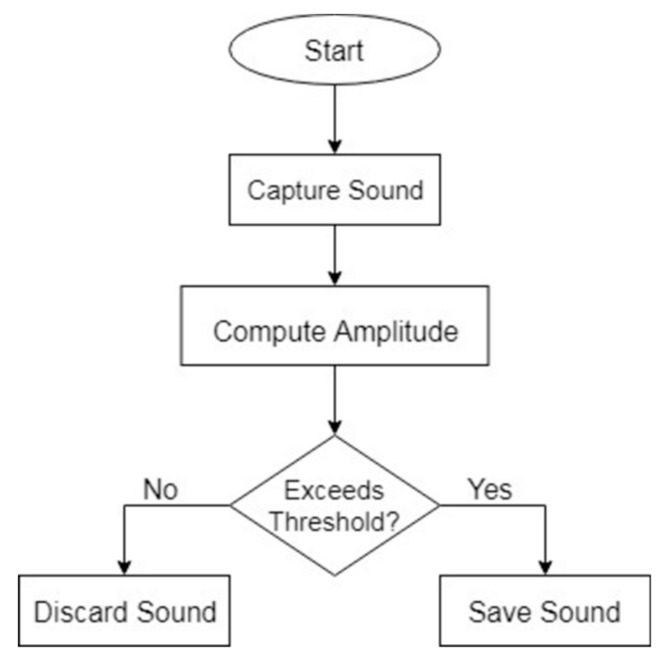
Sound activity detection flow diagram.

During the data collection, a simple sound activity detection step is performed to ensure that the audio files being saved indeed contained sufficient audio signals. This is done by computing the total amplitude of the captured sound and comparing it to a predefined threshold value. The threshold value was determined by observing the total amplitudes of sound files that contained actual signals and setting it close to the minimum observed total amplitude. If a recorded signal’s amplitude is below the threshold value, the sound is discarded, and otherwise it is saved. The sound activity detection algorithm is expressed in both equation and flow diagram forms, as shown in Equation (1) and Figure 3, respectively.

(1) $$decision=\;\left\{\begin{array}{c} save,\;\;\;\;\;\;\;\;\;\;\;\;\;\;if\;A\;\geq \;y\\ discard,\;\;\;\;\;\;\;\;if\;A\;<\;y \end{array}\right.$$

where *A* is computed amplitude, i.e., the sum of squared intensities of the signal samples, and *y* is the predefined threshold value. 

In Table 1, we show the exact number of sounds recorded at each of the nine positions for all the three rooms, and Table 2 also shows the room type, dimensions, volumes, and total number of signals recorded per room. After all the data are recorded and stored, we move to the next stage of transforming them into the appropriate form for the training of our model. The process is explained in Section 2.2.

### 2.2. Data Transformation

**Figure 4:**
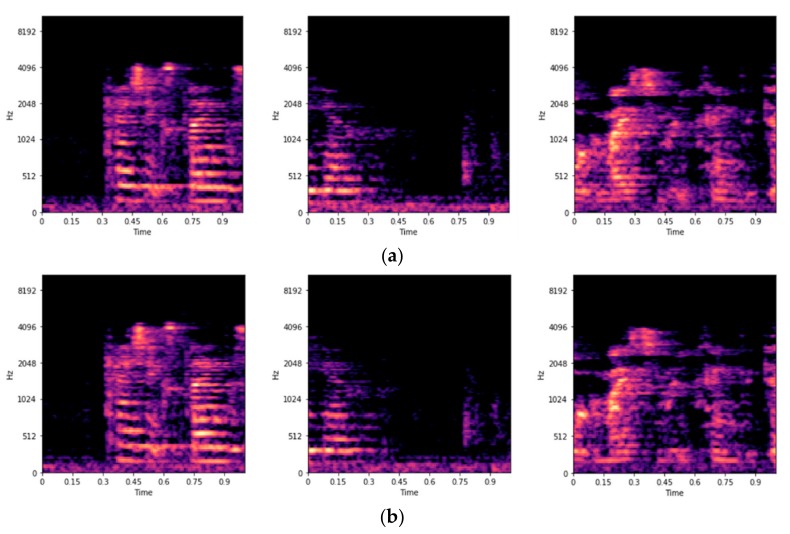
Examples of the log-scaled mel spectrograms extracted from recorded signals. (**a**) Log-scaled mel spectrograms of the first-channel signals; (**b**) Log-scaled mel spectrograms of the second-channel signals.

**Figure 5:**
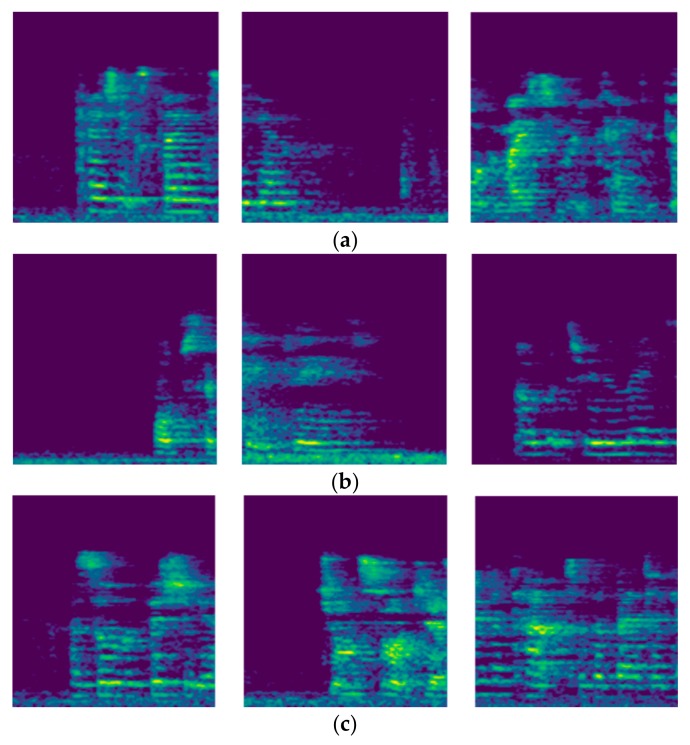
Random selections of the first-channel mel spectrograms. (**a**) Log-scaled mel spectrograms in the one-meter class; (**b**) Log-scaled mel spectrograms in the two-meter class; and (**c**) Log-scaled mel spectrograms in the three-meter class.

The goal of this study is to use a deep learning model to learn relevant distance-related features from the audio signals for classification. To accomplish other deep learning-based audio analysis goals, previous researchers [36,41] have represented the time domain audio signals by mel [33] spectrograms before training. The mel spectrogram is a time-frequency representation of an audio signal that compresses high-frequency components and focuses more on low-frequency components [36]. We use librosa [42] to extract from our dataset log-scaled mel spectrograms of size 128 × 128. Some examples of the log-scaled mel spectrograms of the recorded signals are shown in Figure 4. Along with the transformation, a corresponding ground truth label is saved for each spectrogram. Figure 5 shows some of the first channel mel spectrograms randomly selected from the three distance classes. Given the log-scaled mel spectrograms as our input features, the next step is to design a suitable deep learning model for the training. 

### 2.3. Proposed CRNN Model Architecture

Convolutional neural networks (CNNs) are a powerful type of artificial neural networks that consist of one or more convolutional layers, followed by one or more fully connected layers. The convolutional layers are optionally paired with their respective pooling layers, which enable the extracted features to be downsized appropriately. In 2012, the winner of the ImageNet Large Scale Visual Recognition Challenge (ILSVRC) used a deep CNN model [43], which drew much attention to the outstanding performance of CNNs. Another type of neural network is the recurrent neural network (RNN) [44], which learns by holding on to past memories. In a typical RNN, a unit’s output is influenced by both its own input and the history of previously fed inputs. RNN models are famous for tasks involving sequential data; for example, these include automatic speech recognition tasks [45,46], handwriting recognition tasks [47], and stock price prediction tasks [48]. By stacking these two types of neural networks to form convolutional recurrent neural networks (CRNN), some researchers have achieved remarkable results in acoustic scene analysis and audio processing tasks [36,49].

**Figure 6:**
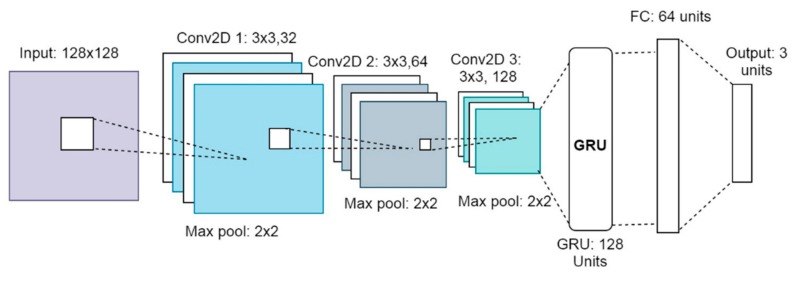
Diagram of the proposed convolutional neural networks (CRNN) model’s architecture.

In this study, a CRNN architecture is employed, which enables the model to learn both the spectral and temporal features and relationships effectively. While the CNN extracts frequency-based information from each input spectrogram by convolution, the RNN analyzes the temporal connections between the extracted feature maps. First, three 2D convolutional layers extract features from the input log-scaled mel spectrograms. The LeakyReLU activation function is applied to the output of each convolution layer, and the activation maps are max-pooled to reduce their dimensions. The downsized feature maps become the input to the next convolution layer. The feature maps from the final convolution layer are reshaped after max-pooling and then passed to the RNN layer. This is specifically a gated recurrent unit (GRU) RNN [50]. Next, the output of the RNN layer is passed to the fully connected layer and then finally to the output layer. The output layer has three neurons, representing the three predefined distance classes. To regularize our model, we applied dropout rates of 25% to the first two convolution layers, 40% to the third convolution layer, and 50% to the fully connected layer. In addition, we applied the L2 weight regularization to all convolution and the recurrent layers using a penalty of 0.001 to further reduce overfitting. Figure 6 shows the architecture of our CRNN model.

## 3. Experiments and Results

The environment used for implementing the proposed method is described as follows. The programs were compiled and executed with the aid of an Intel(R) Core(TM) i7-7700 3.6 GHz CPU PC, in which was installed an NVDIA GeForce 1060 graphics card with a 6 GB frame buffer and an 8 Gbps processing speed. The programming languages used were C++ and Python. To train the model, we used the Keras library [51] with a Tensorflow [52] backend. The training data samples were prepared as described in Section 2.1 and Section 2.2. See Figure 1 and Figure 2 for an illustration of the recording positions. The signals had average signal-to-noise ratios (SNRs) of 3.7 dB, 7.4 dB, and 11.08 dB for room 1, room 2, and room 3, respectively. The noise in each room was first recorded, and afterwards, the signals were played and recorded. The average power of the noise (Power of Noise) for each room was subtracted from the power of the individual recorded sounds to get the power of the wanted signals (Power of Signal). Then, the SNR in decibels (dB) was computed using Equation (2).

(2) $$SNR=10\;\log_{10}\left(\frac{Power\;of\;Signal}{Power\;of\;Noise}\right)\;dB\;$$

**Figure 7:**
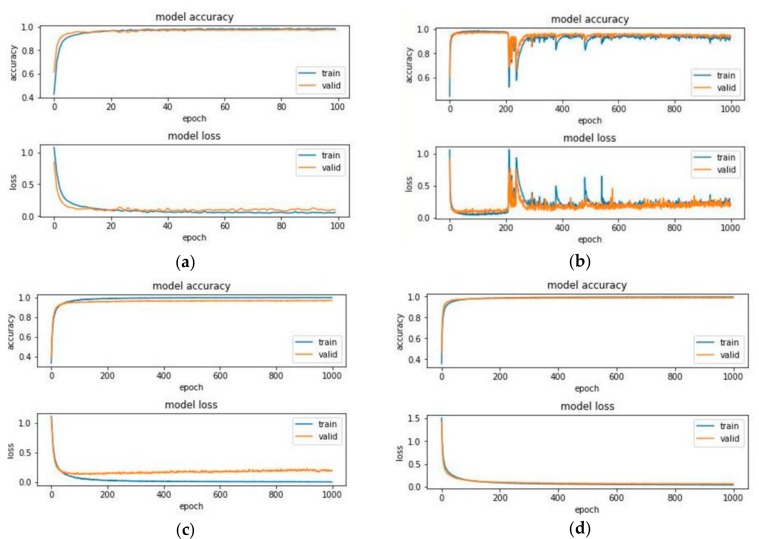
Graphs showing the effect of hyperparameters on the training progress. (**a**) History of the model with 100 epochs, a batch size of 32, and a default learning rate of 0.001; (**b**) History of model with 1000 epochs, a batch size of 32, and a default learning rate of 0.001; (**c**) History of model with 1000 epochs, a batch size of 128, and a learning rate of 0.0001; (**d**) History of model with 1000 epochs, a batch size of 128, a learning rate of 0.0001, and an L2 weight decay value of 0.001.

To begin with, the effects of some of the hyperparameters in our preliminary experiments are discussed as follows. Note that the dropout regularization is already applied to the model. Firstly, an epoch number of 100 was used with a batch size of 32. Figure 7a shows the training history of the model, where we can see that the training and validation accuracies and losses begin to decrease and increase respectively as the training approaches the 100th epoch. Increasing the number of epochs reveals high levels of fluctuations, which can be seen in both the accuracy and loss curves, as shown in Figure 7b. Figure 7c shows some improvement in the learning process after increasing the batch size to 128 and reducing the Adam optimizer’s [53] default learning rate of 0.001 to a rate of 0.0001. This was done in an attempt to stabilize both the accuracy and loss curves. Compared to the Figure 7b curves, the fluctuations are tremendously reduced. Although the training loss values are more stabilized and are reducing consistently, the validation loss values seem to be rather increasing, implying that the model is performing well on the training dataset but is performing poorly on the validation dataset: a classic case of overfitting. To address this problem, L2 weight regularization is applied, and its effect can be seen in Figure 7d. Both the training and validation curves are much similar at this point, and we can see that the curves are very stable. This final configuration of our model was used in all of our experiments presented below.

### 3.1. Classification in Known Environments

In the first experiment, the study sought to train and then test the model in the same environment (i.e., the train dataset and the test dataset are both recorded in the same environment). Multiple trainings were done using our recorded dataset as well as a public dataset. In some cases, the model was trained with the combined dataset from multiple rooms, and in other cases, the model was trained in each room separately.

#### 3.1.1. Our Recorded Dataset

**Figure 8:**
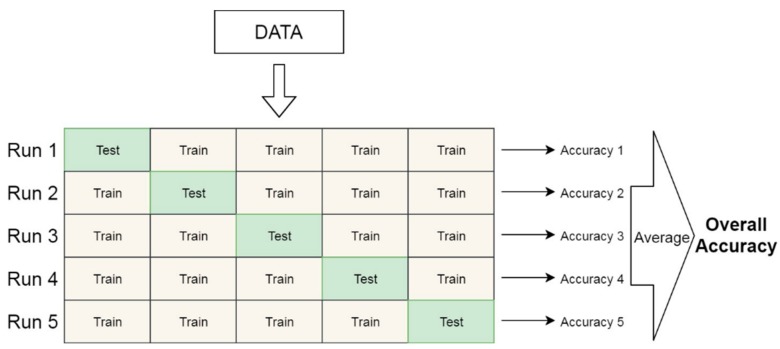
Illustration of the k-fold validation.

In the first case using our dataset, all the data from the three different rooms consisting of approximately 27,000 audio signals were randomly combined to form one dataset and then divided into training and testing sets. A total of 2700 signals were selected from the total dataset—100 signals from each of the nine recording positions in the three rooms. Then, using the stratified k-fold cross-validation [54] approach, the training set consisting of approximately 24,300 was split into k = 5 subsets, and the model was trained five times. Figure 8 illustrates how the k-fold cross-validation works. In each of the five folds, one data subset x is reserved, while the model is trained on the remaining four subsets, and afterwards, it is tested on the reserved subset x. As shown in Figure 8, in every run (fold), a unique subset of data is reserved as the test set, and by the end of the experiments, each of the five subsets of data would have taken its turn as the test set. Table 3 shows the test accuracy of each fold’s model on the test dataset initially reserved, as well as the average accuracy for all five models. 

**Figure 9:**
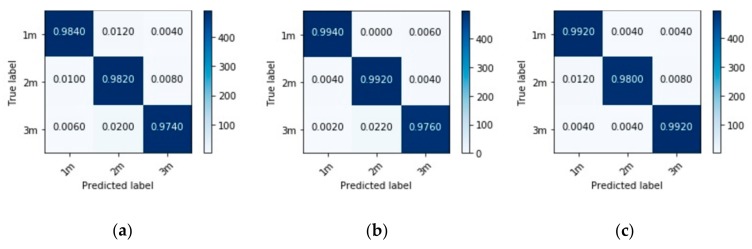
Confusion matrices showing the performances of models trained and tested separately in (**a**) Room 1; (**b**) Room 2; and (**c**) Room 3.

Next, the model is trained and tested in each room separately. First, the dataset for each room is split into training and testing sets, after which the model is trained on the training dataset and evaluated on the testing dataset. The keras function ‘validation_split’ is used to split the training data into training (80%) and validation (20%) sets. In Figure 9, we show the results of evaluating each model on its respective testing set in the form of confusion matrices for performance visualization. In each matrix, each row represents instances in a true class, and each column represents instances in the predicted class. The correctly predicted instances are shown in the diagonal of the matrix, and the values outside the diagonal show the incorrectly predicted instances. Note that utterances from the EUSTACE corpus were repeatedly played and recorded for our dataset; hence, there is similarity in the data, which possibly led to the high classification accuracies recorded in these experiments.

#### 3.1.2. Public Dataset

In the second case, the study sought to examine the performance of the model when trained and then tested on a dataset different from our recorded dataset, which is also a known environment scenario. Using the Aachen room impulse response public dataset [55], we prepared training and testing datasets by convolving with speech data taken from the telecommunications and signal processing (TSP) anechoic speech database [56]. The TSP database consists of over 1400 utterances spoken by 24 speakers. We used utterances from four speakers to prepare the training set, and we used utterances from two separate speakers to prepare a separate test set. The utterances were convolved with impulse responses from four different rooms with different mean reverberation times (RT_60_), as shown in Table 4. The table also shows the various source–microphone distances available in each room as well as their respective distance-specific RT_60_s. The total number of source–microphone distances is 15, and Table 5 shows the class labels for all the 15 classes. After each convolution, we take the first one-second-long segment from the convolved signal, extract the log-scaled mel spectrogram, and then save it as one data sample. 

**Figure 10:**
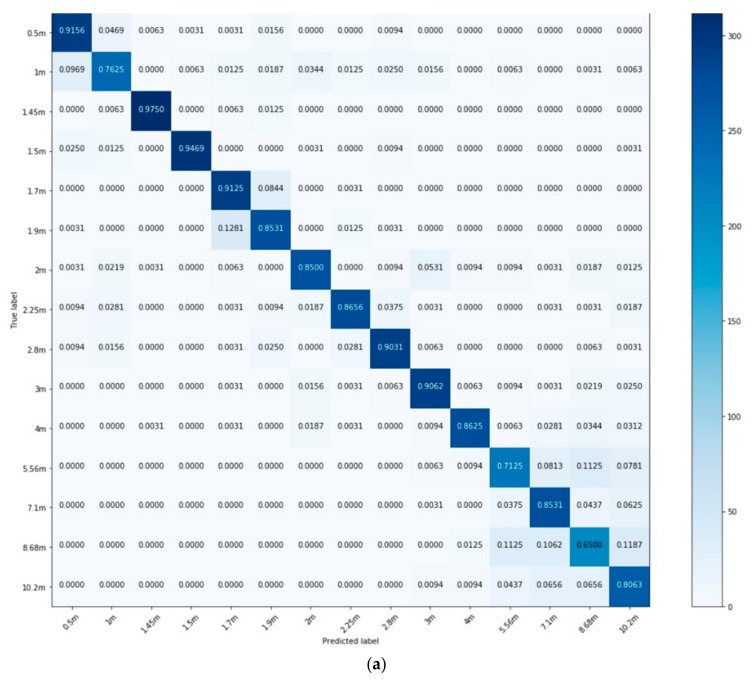

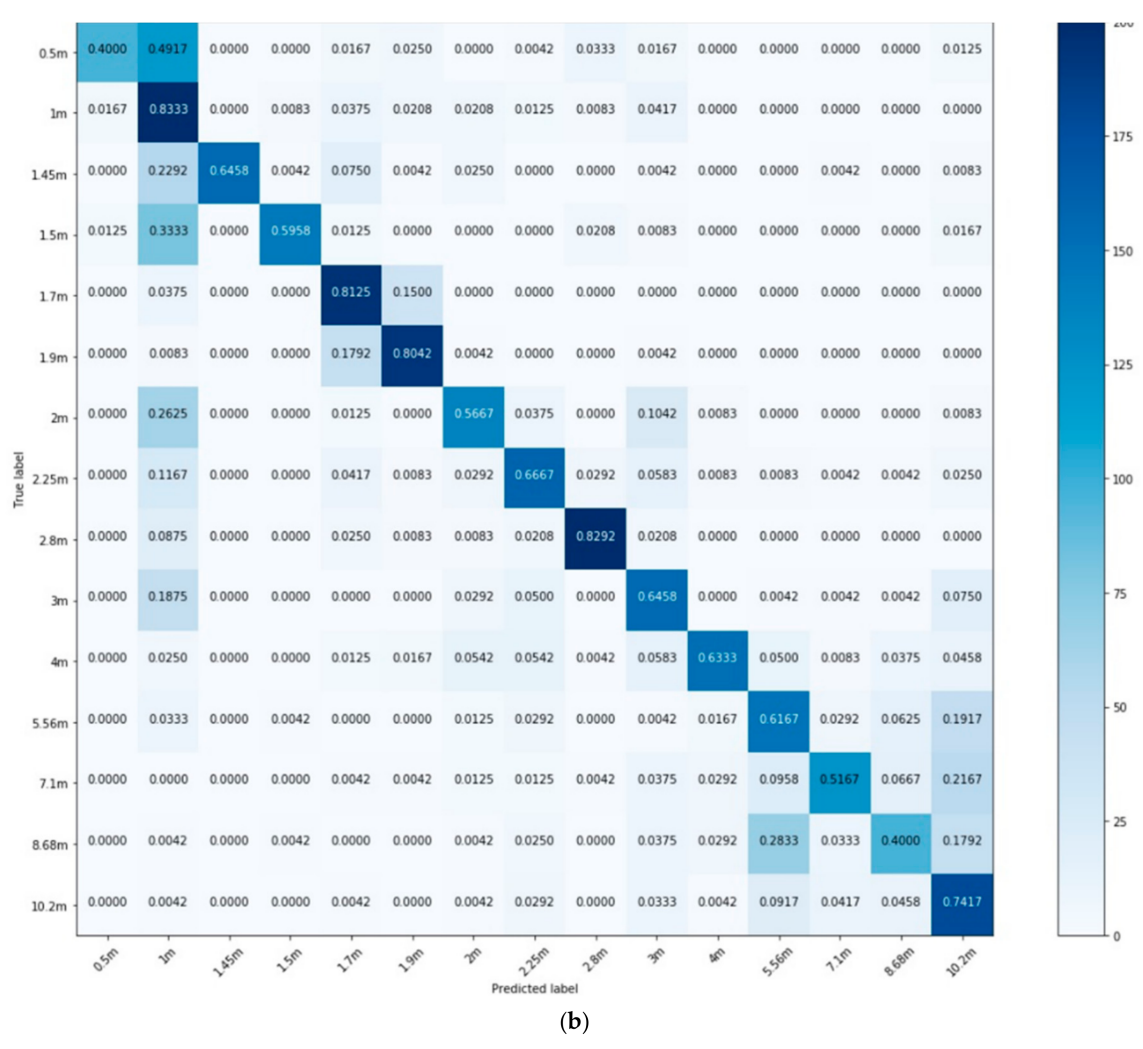
Confusion matrices showing the performances of the model trained on the public dataset. (**a**) Model’s performance on same-speaker test set; (**b**) Model’s performance of different-speaker test set.

The model was trained on the training dataset, using the ‘validation_split’ function to split it into training (80%) and validation (20%) sets. Here, the performance of the model was evaluated on two test sets—a same-speaker test set and a different-speaker test set—and the results are shown in Table 6 and Figure 10. It can be seen both in Table 6 and Figure 10 that the performance of the model on the different-speaker test sets are lower than the performance on the same-speaker test set; however, considering that there are 15 different classes in this experiment, the amount of training data (i.e., 9600) and time (i.e., 1000 epochs) might have been insufficient. Therefore, increasing the training data and time might improve the general performance of this model.

**Figure 11:**
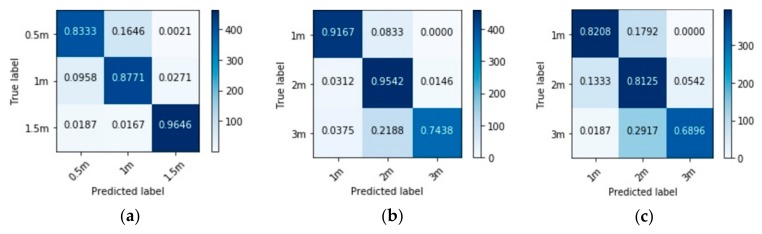

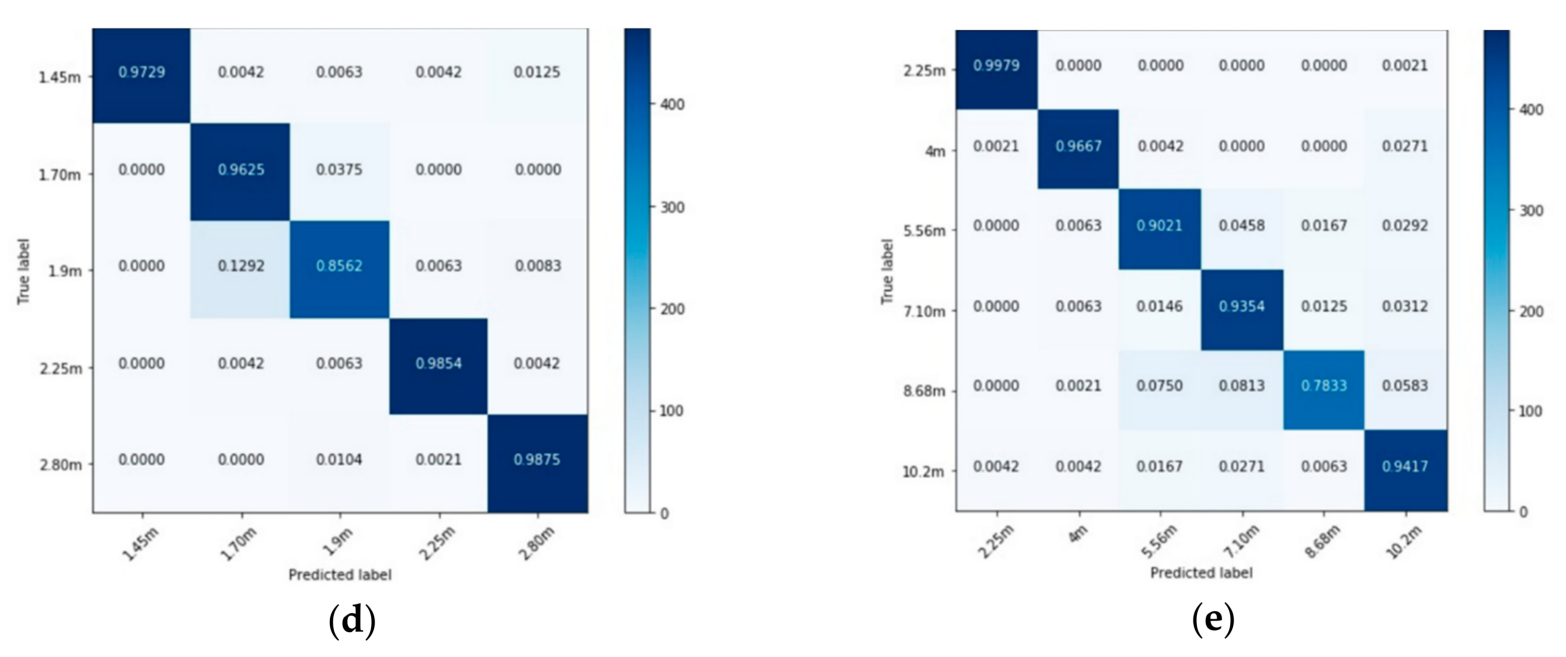
Confusion matrices showing the performances of models trained separately in a (**a**) Studio booth; (**b**) Office room; (**c**) Stairway; (**d**) Meeting room; and (**e**) Lecture room.

Using the public dataset, the model was also trained and tested in each room separately. The utterances used for the training and testing datasets were taken from different speakers; hence, the results presented are all speaker-independent. The performance of each room’s model is shown in the form of a confusion matrix in Figure 11, and their total accuracies are reported in Table 7. 

### 3.2. Classification in Unknown Environments: Generalization Ability

In these experiments, the study sought to train the model in one environment and then test it in a different environment afterwards (i.e., the training dataset and testing dataset are recorded in separate environments).

#### 3.2.1. Our Recorded Dataset

One model was trained for each possible combination of the three rooms, amounting to nine different models. For example, using the room 1–room 2 combination, the model was first trained with all the dataset recorded in room 1, and then the trained model was tested on the dataset recorded in room 2. In other combinations, we combined datasets from two rooms, e.g., room 1 and 2 for the training of the model, and then the model was tested on the dataset from room 3. Table 8 shows the nine different room combinations and the corresponding models’ test accuracies. 

#### 3.2.2. Our Dataset and Public Dataset

In this case, the model was trained with our recorded data and then evaluated on the public dataset. Specifically, we trained the model on our combined dataset (i.e., rooms 1, 2, and 3), and then we tested it on data from the office room and the stairway. These rooms were selected for the evaluation because their distance classes are the same as those in our dataset. Table 9 shows the accuracy of the model’s classification in the two rooms. Again, the classification accuracies are very low, below 50%, which might be due to the difference in the acoustic properties of the training and testing rooms.

### 3.3. Comparison with Previous Method

In this section, we compare the performance of our proposed method to a previous single-channel signal distance estimation method [25]. The method was based on statistical properties of speech source excitation signal being classified into predefined distance classed using a GMM. We compare our method’s accuracies to the accuracies reported in this previous paper. In Table 10, we show the accuracy of our proposed method when trained and tested in an office room with an RT_60_ of 0.43 in comparison to the accuracy of the previous GMM, with the statistics method also trained and tested in a small office room with an RT_60_ of 0.39.

Both models were trained and tested with utterances from different speakers, and it can be seen that the accuracy of our proposed method is much higher. Since it is reported that the GMM with the statistics method performed better in rooms with lower RT_60_, it is possible that if tested in our public data office room (RT_60_ = 0.43), the GMM method’s accuracy will further reduce. However, in terms of method’s robustness when tested on different speaker utterances, the GMM method’s performance remains almost the same and even slightly increases in the room with the lowest RT_60_, whereas our method’s performance slightly reduces when tested on different speakers’ utterances as compared to testing with the same speakers’ utterances. Nonetheless, the reduced accuracy of our model with different speaker test sets is still higher than or comparable to both cases of the GMM with the statistics method.

## 4. Discussion

In this section, the results of the different experiments presented in Section 3 are discussed. In Section 3.1, the experiments demonstrate the performance of the proposed method in known environments. Although the results obtained with our recorded dataset are extremely high, which is probably due to the similarity in the signals, the subsequent results obtained from the Aachen room impulse response [55] datasets confirmed that the proposed method can classify/estimate sound source distances in known environments. In the TSP speech dataset [56] that is convolved with the Aachen room impulse responses, every utterance from a specific speaker is different, and repetitions were avoided by taking only a one-second-long segment of the signal for each convolution. The average accuracy for the five separate public dataset rooms is 88.23%, which was achieved without the need for hand-crafted feature engineering. In comparison with a previous single-channel speaker distance detection method, our proposed method appeared to perform better even with higher RT_60_ rooms, as shown in Section 3.3. This shows that the log-scaled mel spectrograms carry rich information, from which a deep learning model can learn distance-related features. 

To examine the generalizability of the proposed method in unknown environments, various combinations of our recorded datasets from the three rooms were made. The dimensions of the three rooms are specified in Table 2. All three rooms have different dimensions and volumes; however, rooms 1 and 2 are more similar than room 3 in terms of physical structure and the number and location of windows, and the possible effect of this can be seen in the classification accuracies. Table 8 shows the accuracies for all the models trained and tested with the different combinations of datasets. Here, it is observed that the performance of the trained models are reduced significantly compared to known environment scenarios. Especially, in the case of using single room datasets for training and testing, the classification accuracies could fall below 50%. See the results for the room combinations 1 and 3, 3 and 1, and 2 and 3 in Table 8. It was noted that in all the cases involving rooms 1 and 2 as the training and testing room respectively and vice versa, the test accuracy was always above 70%, which may be due to the similarity between rooms 1 and 2, as noted earlier.

Furthermore, training the model with our recordings and testing it on the public dataset resulted in similar low accuracies, as shown in Table 9. The implication of this observation is that the proposed method does not generalize well to unknown or unseen environments, and in order to improve the method’s generalizability, it is essential to expose the neural network to a larger amount of more diverse data samples recorded in more rooms having similar characteristics to those of the expected test or application environments. Moreover, due to the outstanding learning capabilities of deep learning models, it is expected that extending the training of our model by fine-tuning the network and increasing the training time could potentially further improve the model’s performance. In addition, in this study, we considered only single sources, and we will require more data in order to investigate the performance of the model on multiple sound sources. This will be included in the future work of the study.

## 5. Conclusions

This paper proposed a method for estimating the distance of sound sources in rooms by using a convolutional recurrent neural network to classify the audio signals into predefined distance classes. We prepared an audio dataset for the purpose of sound source distance estimation and used log-scaled mel spectrograms of the audio signals as our training input data. The proposed model consisted of three convolution layers, followed by one recurrent layer, a fully connected layer, and an output layer. Our experimental results show that the log-scaled mel spectrogram input features work well in providing adequate distance-dependent information for the network to learn from. Several experiments—grouped into known environments and unknown environments—were performed using the same network architecture and configuration. In addition to our recorded dataset, we used the Aachen room impulse response dataset to prepare more data for the evaluation of our method’s generalization ability, which proved the importance of using relevant or typical data samples to train the neural network. Based on our experimental results, it was concluded that using the log-scaled mel spectrograms, a deep learning model can be trained to accurately classify sounds into the appropriate predefined distance classes, and the method works best in known environments and with similar signal types.

In comparison to other methods, our approach of using time-frequency image representations of the audio signals for training reduces the need for excessive hand-crafted feature engineering. Moreover, due to the remarkable performance of CNN models in image classification tasks, our approach achieves an average accuracy of 88.23% in estimating sound source distances by classification in separate rooms. In our future work, we plan to enlarge our dataset by diversifying it with various kinds of sounds and also applying data augmentation techniques. It is anticipated that in addition to fine-tuning the proposed model, this will have a positive impact on the generalization ability and the accuracy of the model.

## Figures and Tables

**Table 1:** Number of audio signals recorded at each position in all three rooms.

Orientation	Zero Degrees (0°)			Thirty Degrees (30°)			Sixty Degrees (60°)		
Distance (meter)	1 m	2 m	3 m	1 m	2 m	3 m	1 m	2 m	3 m
**Room 1**	1000	993	1000	1000	1000	1000	1000	1000	1000
**Room 2**	1000	1000	945	1000	1000	1000	1000	1000	1000
**Room 3**	1000	1000	1000	1000	1000	1000	1000	1000	1000

**Table 2:** Room types, dimensions, volumes, and total number of recordings per room.

Room	Type	Length (m)	Width (m)	Height (m)	Volume (m^3^)	No. of Signals
Room 1	Research laboratory	8.97	3.45	2.62	81.08	8993
Room 2	Classroom	8.97	7.12	2.62	167.33	8945
Room 3	Computer laboratory	14.8	9.11	2.47	333.03	9000

**Table 3:** Results of fivefold experiments using our recorded datasets combined from all three rooms.

Fold Number	Accuracy (%)
Fold 1	98.51
Fold 2	98.35
Fold 3	98.49
Fold 4	98.58
Fold 5	98.41
Average Accuracy	98.47

**Table 4:** Mean RT_60_, source–microphone distance classes for each of the four rooms and their distance-specific RT_60_ values.

Room	Mean RT_60_ (s)	Source–Microphone Distances (m)	RT_60_ (s)
Studio booth	0.12	0.50	0.08
		1.00	0.11
		1.50	0.18
Office room	0.43	1.00	0.37
		2.00	0.44
		3.00	0.48
Meeting room	0.23	1.45	0.21
		1.70	0.22
		1.90	0.21
		2.25	0.24
		2.80	0.25
Lecture room	0.78	2.25	0.70
		4.00	0.72
		5.56	0.79
		7.10	0.80
		8.68	0.81
		10.2	0.83

**Table 5:** Class labels for each source–microphone distance in the public dataset.

Class	Distance (m)
1	0.5
2	1.0
3	1.45
4	1.5
5	1.7
6	1.9
7	2.0
8	2.25
9	2.8
10	3.0
11	4.0
12	5.56
13	7.1
14	8.68
15	10.2

**Table 6:** Accuracy of the proposed method when trained and tested with the public dataset comprising four different rooms combined.

Test Dataset	Accuracy (%)
Same speakers’ utterances	85.17
Different speakers’ utterances	64.72

**Table 7:** Accuracy of the proposed method when trained and tested in each public dataset room separately.

Test Dataset	Accuracy (%)
Studio booth	89.17
Office room	87.15
Stairway	77.43
Meeting room	95.29
Lecture room	92.12

**Table 8:** Accuracies of the nine different models trained for the various combinations of the three rooms.

Test Number	Training	Testing	Test Accuracy (%)
1	Room 1	Room 2	81.38
2	Room 2	Room 1	74.19
3	Room 1	Room 3	44.82
4	Room 3	Room 1	42.13
5	Room 2	Room 3	48.60
6	Room 3	Room 2	61.61
7	Rooms 1 and 2	Room 3	51.78
8	Rooms 1 and 3	Room 2	86.14
9	Rooms 2 and 3	Room 1	70.17

**Table 9:** Accuracy of the model trained on our recorded dataset and tested on the public dataset.

Training	Test Room	Accuracy (%)
Rooms 1, 2, and 3	Office room	41.67
Rooms 1, 2, and 3	Stairway	38.77

**Table 10:** Comparison of the proposed method to the previous single-channel distance estimation method. GMM: Gaussian mixture model.

Method	RT_60_	Accuracy (%)
Proposed	0.43	87.15
GMM with statistics	0.39	75.4

## References

[1] Murray C.J., Erwin H., Wermter S. Robotic Sound-Source Localization and Tracking Using Interaural Time Difference and Cross-Correlation. Proceedings of the AI Workshop on NeuroBotics.

[2] Wang L., Cavallaro A. Time-Frequency Processing for Sound Source Localization from a Micro Aerial Vehicle. Proceedings of the IEEE International Conference on Acoustics, Speech and Signal Processing.

[3] Chakrabarty S., Habets E.A.P. Broadband DOA Estimation using Convolutional Neural Networks Trained with Noise Signals. Proceedings of the IEEE Workshop on Applications of Signal Processing to Audio and Acoustics.

[4] Ferguson E.L., Williams S.B., Jin C.T. Sound Source Localization in a Multipath Environment Using Convolutional Neural Networks. Proceedings of the IEEE International Conference on Acoustics, Speech and Signal Processing.

[5] Chakrabarty S., Habets E.A.P. Multi-Speaker Localization Using Convolutional Neural Network Trained with Noise. Proceedings of the 31st Conference on Neural Information Processing Systems (NIPS 2017).

[6] Roden R., Moritz N., Gerlach S., Weinzierl S., Goetze S. On Sound Source Localization of Speech Signals using Deep Neural Networks. Proceedings of the 41st Deutsche Jahrestagung fur Akustik Conference (DAGA).

[7] Lee S., Park Y., Park Y. (2015). Three-dimensional Sound Source Localization Using Inter-Channel Time Difference Trajectory. Int. J. Adv. Robot. Syst..

[8] Rodemann T., Ince G., Joublin F., Goerick C. Using Binaural and Spectral Cues for Azimuth and Elevation Localization. Proceedings of the IEEE/RSJ International Conference on Intelligent Robots and Systems.

[9] Perotin L., Serizel R., Vincent E., Guerin A. CRNN-Based Joint Azimuth and Elevation Localization with the Ambisonics Intensity Vector. Proceedings of the 16th International Workshop on Acoustic Signal Enhancement (IWAENC).

[10] Frejlichowski D., Gosciewska K., Forczmanski P., Hofman R. (2014). “SmartMonitor”—An Intelligent Security System for the Protection of Individuals and Small Property with the Possibility of Home Automation. Sensors.

[11] Djahel S., Smith N., Wang S., Murphy J. Reducing emergency services response time in smart cities: An advanced adaptive and fuzzy approach. Proceedings of the IEEE First International Smart Cities Conference.

[12] Meza I., Rascon C., Fuentes G., Pineda L.A. (2016). On Indexicality, Direction of Arrival of Sound Sources and Human–Robot Interaction. J. Robot..

[13] Do H.M., Sheng W., Liu M. (2016). Human-assisted sound event recognition for home service robots. Robot. Biomim..

[14] Zhang T., Mustiere F., Micheyl C. Intelligent Hearing Aids: The Next Revolution. Proceedings of the 38th Annual International Conference of the IEEE Engineering in Medicine and Biology (EMBC).

[15] Lu C., Wu C., Fu C. (2011). A reciprocal and Extensible architecture for multiple-target tracking in a Smart Home. IEEE Trans. Syst. Man Cybern. Part C Appl. Rev..

[16] Sylvain A., Patrick D., Philippe S. (2015). A Survey on Sound Source Localization in Robotics: From Binaural to Array Processing Methods. Comput. Speech Lang..

[17] Samarasinghe P.N., Abhayapala T.D., Polettfi M.A., Betlehem T. On Room Impulse Response between Arbitrary Points: An Efficient Parameterization. Proceedings of the 6th International Symposium on Communication, Control and Signal Processing (ISCCSP).

[18] Bronkhorst A.W. Modeling Auditory Distance Perception in Rooms. Proceedings of the AAE Forum Acusticum.

[19] Chen H., Abhayapala T.D., Samarasinghe P.N., Zhang W. (2017). Direct-to-Reverberant Energy Ratio Estimation using a First-Order Microphone. IEEE/ACM Trans. Audio Speech Lang. Process..

[20] Lu Y.C., Cooke M. (2010). Binaural Estimation of Sound Source Distance via the Direct-to-Reverberant Energy Ratio for Static and Moving Sources. IEEE Trans. Audio Speech Lang. Process..

[21] Rodemann T. A Study on Distance Estimation in Binaural Sound Localization. Proceedings of the IEEE/RSJ International Conference on Intelligent Robots and Systems.

[22] Honda S., Shinohara T., Uebo T., Nakasako N. Estimating the Distance to a Sound Source using Single-Channel Cross-Spectral Method between Observed and Pseudo-Observed Waves based on Phase Interference. Proceedings of the 23rd International Congress on Sound & Vibration.

[23] Vesa S. (2009). Binaural Sound Source Distance Learning in Rooms. IEEE Trans. Audio Speech Lang. Process..

[24] Georganti E., May T., Par S.V.D., Mourjopoulos J. (2013). Sound Source Distance Estimation in Rooms based on Statistical Properties of Binaural Signals. IEEE Trans. Audio Speech Lang. Process..

[25] Georganti E., May T., Par S.V.D., Harma A., Mourjopoulos J. (2011). Speaker Distance Detection using a Single Microphone. IEEE Trans. Audio Speech Lang. Process..

[26] Niu H., Reeves E., Gerstoft P. (2017). Source localization in an ocean waveguide using supervised machine learning. J. Acoust. Soc. Am..

[27] Brendel A., Kellermann W. Learning–based acoustic source –microphone distance estimation using the coherent-to-diffuse power ratio. Proceedings of the IEEE International Conference on Acoustics, Speech and Signal Processing.

[28] Huang Z., Xu J., Gong Z., Wang H., Yan Y. (2019). Multiple source localization in a shallow water waveguide exploiting subarray beamforming and deep neural networks. Sensors.

[29] Niu H., Gong Z., Ozanich E., Gerstoft P., Wang H., Li Z. (2019). Deep-learning source localization using multi-frequency magnitude-only data. J. Acoust. Soc. Am..

[30] Yiwere M., Rhee E.J. (2017). Distance Estimation and Localization of Sound Source in Reverberant Conditions using Deep Neural Networks. Int. J. Appl. Eng. Res..

[31] Tang D., Qin B., Liu T. Document Modeling with Gated Recurrent Neural Network for Sentiment Classification. Proceedings of the 2015 Conference on Empirical Methods in Natural Language Processing.

[32] Zuo Z., Shuai B., Wang G., Liu X., Wang X., Wang B., Chen Y. Convolutional Recurrent Neural Networks: Learning Spatial Dependencies for Image Representation. Proceedings of the IEEE Conference on Computer Vision and Pattern Recognition Workshops (CVPRW).

[33] Stevens S.S., Volkmann J., Newman E.B. (1937). A scale for the measurement of the psychological magnitude pitch. J. Acoust. Soc. Am..

[34] Harma A. Ambient telephony: Scenarios and research challenges. Proceedings of the 8th Annual Conference of the International Speech Communication Association (INTERSPEECH).

[35] McLoughlin I., Zhang H., Xie Z., Song Y., Xiao W. (2015). Robust Sound Event Classification Using Deep Neural Networks. IEEE/ACM Trans. Audio Speech Lang. Process..

[36] Lim H., Park J., Lee K., Han Y. Rare Sound Event Detection using 1D Convolutional Recurrent Neural Networks. Proceedings of the Detection and Classification of Acoustic Scenes and Events 2017 Workshop.

[37] Graves A., Mohamed A., Hinton G. Speech Recognition with Deep Recurrent Neural Networks. Proceedings of the IEEE International Conference on Acoustics Speech, and Signal Processing.

[38] He W., Motlicek P., Odobez J. Deep Neural Networks for Multiple Speaker Detection and Localization. Proceedings of the IEEE International Conference on Robotics and Automation (ICRA).

[39] White L.S., King S. (2013). The EUSTACE Speech Corpus. http://www.cstr.ed.ac.uk/projects/eustace.

[40] Bencina R., Burk P. PortAudio—An Open Source Cross Platform Audio API. Proceedings of the International Computer Music Conference (ICMC).

[41] Pandeya Y.R., Kim D., Lee J. (2018). Domestic Cat Sound Classification using Learned Features from Deep Neural Nets. Appl. Sci..

[42] McFee B., Raffel C., Liang D., Ellis D.P.W., McVicar M., Battenberg E., Nieto O. Librosa: Audio and Music Signal Analysis in Python. Proceedings of the 14th Python in Science Conference.

[43] Krizhevsky A., Sutskever I., Hinton G. ImageNet Classification with Deep convolutional Neural Networks. Proceedings of the Advances in Neural Information Processing Systems 25: 26th Annual Conference on Neural Information Processing Systems.

[44] Parascandolo G., Huttunen H., Virtanen T. Recurrent Neural Networks for Polyphonic Sound Event Detection in Real Life Recordings. Proceedings of the IEEE International Conference on Acoustics Speech and Signal Processing.

[45] Miao Y., Gowayyed M., Metze F. EESEN: End-to-End Speech Recognition using Deep RNN Models and WFST-Based Decoding. Proceedings of the 2015 IEEE Workshop on Automatic Speech Recognition and Understanding (ASRU).

[46] Park J., Boo Y., Choi I., Shin S., Sung W. Fully Neural Network Based Speech Recognition on Mobile and Embedded Devices. Proceedings of the 32nd Conference on Neural Information Processing Systems.

[47] Graves A., Schmidhuber J. Offline Handwritting Recognition with Multidimensional Recurrent Neural Networks. Proceedings of the 22nd Conference on Neural Information Processing Systems.

[48] Nelson D.M.Q., Pereira A.C.M., de Oliveira R.A. Stock Market’s Price Movement Prediction with LSTM Neural Networks. Proceedings of the International Joint Conference on Neural Networks (IJCNN).

[49] Adavanne S., Politis A., Nikunen J., Virtanen T. (2019). Sound Event Localization and Detection of Overlapping Sources using Convolutional Recurrent Neural Networks. IEEE J. Sel. Top. Signal Process..

[50] Cho K., Merrienboer B.V., Gulcehre C., Bahdanau D., Bougares F., Schwenk H., Bengio Y. Learning Phrase Representation using RNN Encoder-Decoder for Statistical Machine Translation. Proceedings of the Conference on Empirical Methods in Natural Language Processing.

[51] Chollet F. Keras. https://github.com/fchollet/keras.

[52] Abadi M., Agarwal A., Barham P., Brevdo E., Chen Z., Citro C., Corrado G.S., Davis A., Dean J., Devin M. (2016). TensorFlow: Large-scale machine learning on heterogeneous systems. arXiv.

[53] Kingma D.P., Ba J.L. Adam: A Method for Stochastic Optimization. Proceedings of the International Conference on Learning Representations.

[54] Kohavi R. A study of cross-validation and bootstrap for accuracy estimation. Proceedings of the International Joint Conference on Artificial Intelligence.

[55] Jeub M., Schafer M., Vary P. A binaural room impulse response database for the evaluation of dereverberation algorithms. Proceedings of the 16th International Conference on Digital Signal Processing.

[56] Kabal P. (2018). TSP Speech Database.

